# HIV-1 Molecular Epidemiology, Transmission Clusters and Transmitted Drug Resistance Mutations in Central Brazil

**DOI:** 10.3389/fmicb.2019.00020

**Published:** 2019-01-31

**Authors:** Tayana Serpa Ortiz Tanaka, Thaysse Ferreira Leite, Solange Zacalusni Freitas, Gabriela Alves Cesar, Grazielli Rocha de Rezende, Andrea De Siqueira Campos Lindenberg, Monick Lindenmeyer Guimarães, Ana Rita Coimbra Motta-Castro

**Affiliations:** ^1^Programa de Pós-graduação em Doenças Infecciosas e Parasitárias, Faculdade de Medicina, Federal University of Mato Grosso do Sul, Campo Grande, Brazil; ^2^Laboratory of AIDS and Molecular Immunology, Oswaldo Cruz Institute, Oswaldo Cruz Foundation, Rio de Janeiro, Brazil; ^3^Universitary Hospital Maria Aparecida Pedrossian, HUMAP, Federal University of Mato Grosso do Sul, Campo Grande, Brazil; ^4^Laboratory of Clinical Immunology, FACFAN, Federal University of Mato Grosso do Sul, Campo Grande, Brazil; ^5^Laboratory of Molecular Virology, Oswaldo Cruz Foundation, Mato Grosso do Sul, Campo Grande, Brazil

**Keywords:** HIV, MSM, molecular epidemiology, transmitted drug resistance, transmission network

## Abstract

We aimed to characterize HIV-1 molecular epidemiology and transmission clusters among heterosexual (HET) and men who have sex with men (MSM) individuals, as well as transmitted drug resistance mutations (TDRM) in Central-Western Brazil. This cross-sectional survey was conducted among 190 antiretroviral naïve HIV-1 infected individuals. Proviral DNA was extracted, and nested PCR amplified partial *polymerase* gene (PR/RT). After sequencing, subtypes were assigned, and the sequences were analyzed for the occurrence of possible transmission networks. Calibrated Population Resistance (CPR) tool from Stanford HIV Database was used to investigate the presence of TDRM. Among 150 individuals whose samples were successfully sequenced, the most prevalent HIV-1 subtype was B, followed by recombinant forms. The occurrence of twenty transmission clusters composed by at least two sequences was verified, suggesting the existence of transmission clusters among individuals from the same or distinct sexual orientations. Intermediate level of TDRM (12%) was found in the study population, and almost half of the subjects with TDRM had more than one resistance mutation. No correlations between sexual orientation and the presence of TDRM, HIV-1 subtypes/recombinants forms were verified. Taken together, the necessity of the continuous monitoring of the TDRM to verify the importance of pre-genotyping and to delineate future strategies in primary antiretroviral therapy. Likewise, the knowledge of the HIV-1 transmission networks in Brazil would allow the implementation of effective HIV-1 prevention strategies in local settings.

## Introduction

In Latin America, it is estimated that 1.8 million people are living with human immunodeficiency virus (HIV) and/or acquired immunodeficiency syndrome (AIDS). Despite 100,000 new HIV infections having been diagnosed in 2017, the HIV incidence decreased 13.7% between 2000 and 2017 (UNAIDS, 2018). In Brazil, HIV prevalence among the general population is below 0.6% and it is estimated that AIDS cases among Brazilians reached 882,810 by June 2017 (Brasil, 2017). HIV prevalence is higher in key populations at risk, for example 17.5% in men who have sex with men (MSM) (Kerr et al., 2018). The detection rate of AIDS has been falling steadily in Brazil in recent years. However, the Central Western region showed little change in its detection rate in the last 10 years, reaching 16.7 cases per 100 thousand inhabitants in 2016 (Brasil, 2017).

Universal access to combined antiretroviral therapy (cART) in Brazil was crucial in order to increase survival and decrease AIDS-related hospitalizations in HIV-1 infected individuals (Souza Junior et al., 2011). Although, the development of drug resistance mutations is a significant obstacle to maintaining HIV-1 replication suppression and can lead to viral load increase and consequently transmission of viruses with drug resistance mutations. Therefore, transmitted drug resistance mutations (TDRM) have become an important challenge, since they have been described for all drugs used in the clinical management of HIV and as incidence and prevalence vary by region this highlights the importance of its monitoring. The prevalence of TDRM could vary according to the study population, methods and lists of resistance mutations used to calculate these rates (Booth and Geretti, 2007).

Brazil has an extensive border, covering about 15,000 km, exhibiting great socioeconomic and cultural diversity across regions. Concerning HIV-1 subtypes, subtype B is the most prevalent, followed by F1, and BF1 recombinants in most Brazilian regions (De Sa Filho et al., 2005; Pedroso et al., 2007; Machado et al., 2009; Guimarães et al., 2015), except for the Southern region, where subtype C is highly prevalent (Silva et al., 2010; de Medeiros et al., 2011; Gräf et al., 2011). However, even in the same geographic region, the HIV-1 distribution could be heterogeneous (Gräf and Pinto, 2013). In border areas, intense drug trafficking and prostitution occur; both situations may affect local epidemic dynamics. Taking these geographical and epidemiological characteristics together into consideration, the study of HIV-1 genetic diversity and transmission networks as well as drug resistance mutations in this region is relevant.

## Materials and Methods

### Subjects and Study Design

We conducted a cross-sectional survey among antiretroviral naïve HIV-infected individuals recruited in Campo Grande, the capital of Mato Grosso do Sul (MS) State, from 2011 to 2014. One hundred and seventy-two individuals were enrolled at Reference Centers for Parasitic and Infectious Diseases (Freitas et al., 2014), and thirty-two were MSM recruited in a cross-sectional study (Fernandes et al., 2015). Inclusion criteria were: (a) having confirmed diagnosis for HIV-1; (b) being over 18 years old; (c) being antiretroviral naïve; (d) having signed the informed consent form in earlier surveys, which predicted storage of samples and their utilization in future research; and (e) having sample stored in sufficient quantity to perform the analyses proposed. Following these criteria, 190 individuals were selected for the subsequent analysis. This study was carried out in accordance with the recommendations of the Ethical Committee on Human Research of the Federal University of Mato Grosso do Sul, that is in accordance with the Declaration of Helsinki. The protocol was approved by under protocol number 1151451, CAAE 46185915.8.0000.0021.

### Amplification of HIV-1 PR/RT Region

DNA was extracted from 200 μL of each whole blood sample by using the QIAamp DNA Blood Mini kit (Qiagen, Hilden, Germany) according to the manufacturer’s protocol. The partial *polymerase* (*pol*) gene including protease/reverse transcriptase (PR/RT) region was amplified by nested polymerase chain reaction (PCR) using combinations of primers described elsewhere (Delatorre et al., 2017). The amplified products were analyzed by electrophoresis using agarose gels (1%). Amplicons were purified using the Illustra GFX^®^ PCR DNA and Gel Band Purification Kit (GE Healthcare, United Kingdom), following the manufacturer’s recommendations. The purified DNA was sequenced using Big Dye Terminator Cycle Sequencing Ready Reaction kit v.3.1 (Applied Biosystems, CA, United States) and processed with an automated ABI 3130xl sequencer (Applied Biosystems), using Sanger’s method.

### Sequence Analysis

The sequences were edited in DNASTAR software and then aligned with reference sequences from Los Alamos HIV Sequence Database^1^ using the Clustal W program implemented in MEGA 6.0 software (Tamura et al., 2013). All sequences are available in GenBank (accession number MF545192-MF545340). The final PR/RT alignment covered a fragment of 1261 bp, corresponding to nucleotides 2254 to 3514 relative to the HXB2 genome.

Maximum Likelihood (ML) phylogenetic was constructed with the PhyML 3.0 program using an online web server (Guindon et al., 2010). The Smart Model Selection recommended the GTR+I+G nucleotide substitution model to be used in the ML (Lefort et al., 2017). The heuristic tree search was performed using the SPR branch-swapping algorithm, and the branch support was calculated with the approximate likelihood-ratio (aLRT) SH-like test (Anisimova and Gascuel, 2006). Recombinant profiles were inferred by bootscan analyses with a sliding window of 300 bp, steps of 10 bp and Kimura-2 parameters model using SimPlot 3.5.1 software (Lole et al., 1999).

Those sequences that clustered together with high aLRT support (>0.90) in the ML tree were analyzed for the occurrence of possible transmission clusters. Therefore, such sequences were submitted to analysis using nucleotide Basic Local Alignment Search Tool (BLASTn) (Altschul et al., 1990) to recover reference sequences with high similarity (>95%). These sequences retrieved were added to three new alignments from pure subtypes (B, D, and F1), and a new ML tree was obtained to verify the maintenance of the transmission clusters according to their subtypes. For subtypes D and F1 analyses we included all available Brazilian reference sequences, however, duplicate sequences were removed. For subtype B, at least ten representative sequences from each Brazilian State and all sequences from Mato Grosso do Sul state available at the Los Alamos HIV Sequence Database were included. Before performing the phylogenetic analyses to confirm the transmission clusters, drug-resistance mutations positions were stripped from each alignment, resulting in a fragment of 891 bp from nucleotides 2262 to 3251 relative to HXB2 genome. Our final cluster classification was defined based on aLRT (>90) in the phylogenetic analyses (Figure 2, 3), and low mean pairwise genetic distances (≤4.5) of clustered sequences have been employed.

### Genotypic Analysis of HIV-1 Drug Resistance

To investigate the presence of TDRM, the sequences were submitted to Stanford HIV Database for Transmitted DRM [TDRM/Calibrated Population Resistance Tool (CPR Tool)] Version 6.0 (Gifford et al., 2009), which uses the mutation list according to Bennett et al. (2009).

### Statistical Analysis

Statistical analyses were conducted using the SPSS 17.0 statistical analysis software package (SPSS Inc., Chicago, IL, United States). Median, standard deviation (SD), range and frequencies (%) were used to describe patients’ characteristics. The frequency of TDRMs was also calculated, and the chi-square or Fisher exact test was employed when appropriate. A *p* value of < 0.05 was defined as statistically significant.

## Results

Out of 190 antiretroviral naïve patients who had samples available for DNA extraction, 172 were PR/RT amplified (90.5%), and from them 150 (87.2%) were successfully sequenced. From those 150 studied subjects, 62.0% were male, with an average age of 36 years, ranging from 18 to 70 years. More than half of participants were white (53.3%), heterosexual (64.0%), and reported less than 12 years of schooling (80.7%), and irregular condom use (54%). Only 6.7% of them were sex workers. Sociodemographic and behavioral characteristics are listed in Table 1. No statistically significant correlation was detected between the variables presented in Table 1 and HIV-1 subtypes.

**Table 1:** Sociodemographic and behavioral characteristics of 150 cART-naïve subjects according to the HIV-1 most frequent subtypes, Central Brazil.

Variable	N	(%)	Subtype B	Sub-subtype F1	Subtype C
**Gender**					
Male	93	(62.0)	64 (63.4)	10 (66.7)	7 (58.3)
Female	57	(38.0)	37 (36.6)	5 (33.3)	5 (41.7)
Age (years)					
18–29	49	(32.7)	33 (32.7)	3 (20.0)	6 (50.0)
30–39	51	(34.0)	36 (35.6)	8 (53.3)	2 (16.7)
40 or more	50	(33.3)	32 (31.7)	4 (26.7)	4 (33.3)
**Skin color/ethnicity**					
White	80	(53.3)	57 (56.4)	8 (53.3)	6 (50.0)
Non-white	70	(46.7)	44 (43.6)	7 (46.7)	6 (50.0)
**Educational (years)**					
0	4	(2.7)	3 (3.0)	0 (0.0)	0 (0.0)
1–12	117	(78.0)	79 (78.2)	11 (73.3)	9 (75.0)
≥12	29	(19.3)	19 (18.8)	4 (26.7)	3 (25.0)
**Monthly income**					
<2 minimum wages	20	(13.3)	11 (10.9)	1 (6.7)	2 (16.7)
2–5 minimum wages	97	(64.7)	65 (64.3)	12 (80.0)	6 (50.0)
>5 minimum wages	31	(20.7)	24 (23.7)	2 (13.3)	4 (33.3)
Missing	2	(1.3)	1 (0.1)	0 (0.0)	0 (0.0)
**Frequency of alcohol consumption**					
None	92	(60.9)	63 (62.4)	8 (53.3)	7 (58.3)
Weekly	51	(35.1)	34 (33.7)	6 (40.0)	3 (25.0)
Daily	7	(4.0)	4 (3.9)	1 (6.7)	2 (16.7)
**Illicit drug use**					
No	118	(78.7)	80 (79.2)	11 (73.3)	8 (66.7)
Yes, no injecting drugs	28	(18.6)	18 (17.8)	3 (20.0)	4 (33.3)
Yes, injecting drugs	4	(2.7)	3 (3.0)	1 (6.7)	0 (0.0)
Sexual orientation					
Heterosexual, female	57	(38.0)	37 (36.6)	5 (33.3)	5 (41.7)
Heterosexual, male	39	(26.0)	27 (26.8)	3 (20.0)	3 (25.0)
MSM	54	(36.0)	37 (36.6)	7 (46.7)	4 (33.3)
**Number of sexual partners in the last 12 months**					
0	13	(8.7)	6 (5.9)	2 (13.2)	1 (8.3)
1	77	(51.3)	53 (52.5)	7 (46.7)	6 (50.0)
2–5	40	(26.7)	28 (27.7)	4 (26.7)	4 (33.4)
6–10	4	(2.7)	3 (3.0)	1 (6.7)	0 (0.0)
>10	16	(10.6)	11 (10.9)	1 (6.7)	1 (8.3)
**Use of condoms in the last 12 months**					
Always	69	(46.0)	51 (50.5)	6 (40.0)	6 (50.0)
Occasionally/Never	81	(54.0)	50 (49.5)	9 (60.0)	6 (50.0)
**Presence of TDRM**					
Yes	18	(12.0)	14 (13.9)	0 (0.0)	2 (16.7)
No	132	(88.0)	87 (86.1)	15 (100)	10 (83.3)


*MSM, men who have sex with men; TDRM, transmitted drug resistance mutations.*

**FIGURE 1:**
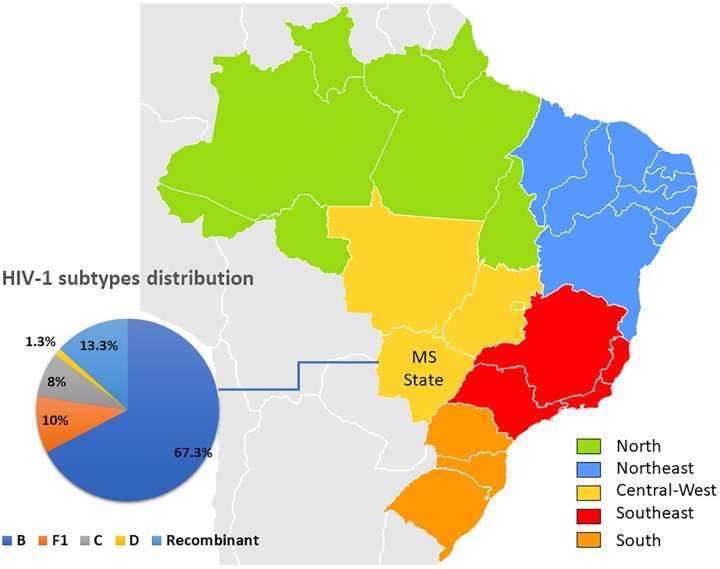
Map of Brazil indicating Brazilian regions and MS State, from which HIV-1 sequences were obtained. The pie chart shows HIV-1 subtypes distribution based on *pol* sequences included in this study.

**FIGURE 2:**
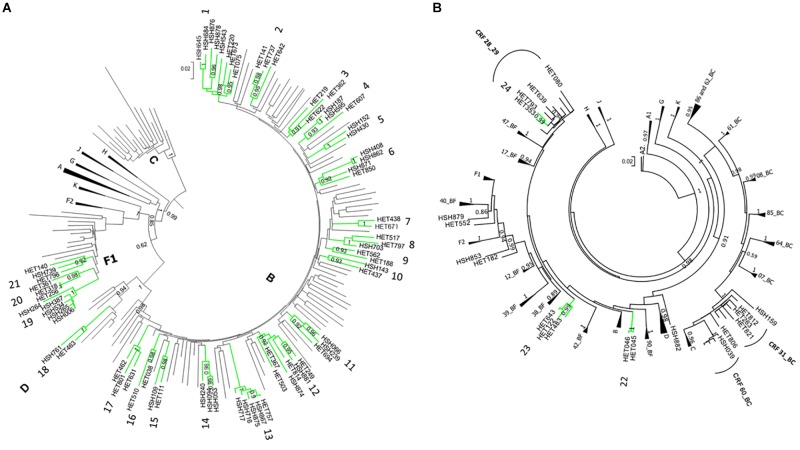
ML phylogenetic tree of 150 HIV-1 PR/RT sequences from Mato Grosso do Sul, Central-West Brazil. The analyzed PR/RT alignment covered a fragment of 1261 bp, corresponding to nucleotides 2254 to 3514 relative to HXB2 genome. Reference sequences retrieved from GenBank are not labeled. ALRT values are represented only if greater > 0.90. Possible HIV-1 transmission clusters were indicated by numbers. HET = sample obtained from heterosexual individual; HSH = sample obtained from men who have sex with men. **(A)** Pure HIV-1 subtypes and **(B)** Recombinant sequences.

Phylogenetical analyses revealed that 101 sequences (67.3%) were classified as subtype B, 15 (10%) as F1, 12 (8%) as C, two (1.3%) as subtype D and 20 (13.3%) possible recombinants (Figure 1, 2A). The phylogenetic and bootscan analysis of these twenty sequences revealed that four (2.7%) were CRF28_29BF, three (2.0%) were CRF31_BC, one (0.7%) was CRF60_BC, and 12 (8.0%) were unique recombinant forms (URF) (Figure 2B).

TDRM to at least one class of antiretroviral drug was found in 18 sequences (12%), and the drug resistance mutation to nucleoside reverse transcriptase inhibitor (NRTI) was the most common (12/150; 8%), followed by non-nucleoside reverse transcriptase inhibitor (NNRTI) (7/150; 4.7%) and PI resistance (3/150; 2%) (Table 2). Of these, twelve (8%) were singleton mutations and six (4.0%) multiple. K103N was the most frequent resistance mutation observed (5/150; 3.3%) followed by V75M (4/150; 2.7%). There was no statistical difference between sexual orientation and the prevalence of TDRM and HIV-1 subtypes distribution.

**Table 2:** Characteristics of the 18 cART-naïve subjects with TDRM.

ID	Age/Gender	Resistance mutations			HIV-1 Subtype	Co-infection
				
		NRTI	NNRTI	PI		
HET080	50/F	–	V106M	–	BF1	Lifetime syphilis^a^
HET116	23/M	–	K103N	M46I	B	
HSH187	31/M	V75M	–	N88D	B	Lifetime syphilis^a^
HSH430	40/M	L210W, T215D	–	–	B	Hepatitis B^b^
HET446	31/F	–	V106M	–	B	–
HET463	19/M	–	K103N	–	D	–
HSH502	27/M	D67N, K219Q	–	–	C	Lifetime syphilis^a^
HET510	40/F	M41L, T215D	–	M46I, V82T, L90M	B	–
HET521	26/F	K70R	–	–	B	–
HET545	40/M	–	K103N	–	B	Lifetime syphilis^a^ Hepatitis B^b^
HET573	37/F	V75M	–	–	B	–
HSH595	28/M	V75M	–	–	B	Hepatitis B^b^
HET607	32/M	V75M	–	–	B	–
HET809	29/M	F77L	–	–	C	–
HET810	31/F	T215S	–	–	B	–
HSH851	21/M	L210W	–	–	B	–
HSH876	27/M	–	K103N	–	B	Lifetime syphilis^a^ Hepatitis B^b^
HSH878	22/M	M184V	K103N, P225H	–	B	Lifetime syphilis^a^


*HET, sample obtained from heterosexual individual; HSH, sample obtained from men who have sex with men; ID, sample identification; NNRTI, Non-Nucleoside Reverse Transcriptase Inhibitor; NRTI, Nucleoside Reverse Transcriptase Inhibitor; PI, Protease inhibitor. ^*a*^Lifetime syphilis: anti-*Treponema pallidum* positivity in ELISA. ^*b*^Hepatitis B: anti-HBc total and/or HBsAg seroposivity in ELISA.*

**FIGURE 3:**
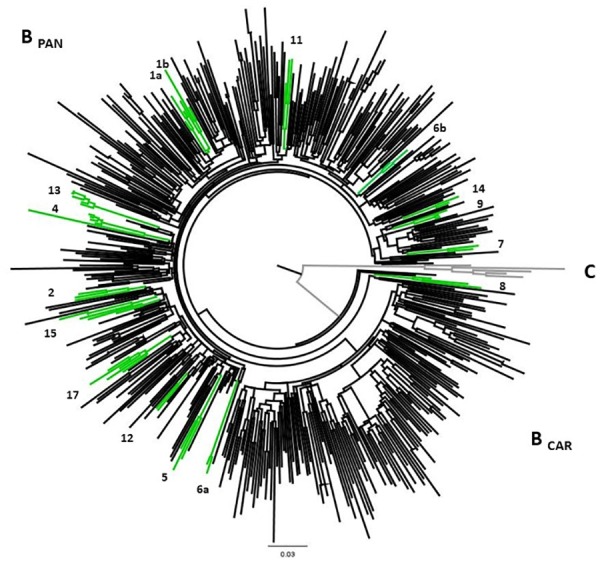
ML phylogenetic tree highlighting HIV-1 subtype B transmission clusters. The confirmed HIV-1 transmission clusters were highlighted in green and numbered according to the previous grouping from Figure 1. All clusters present aLRT ≥ 0.90 and low mean pairwise genetic distances (≤4.5). The analysis involved 520 HIV-1 B PR/RT sequences (102 sequences from the present study, 331 Brazilian reference sequences, 82 non-Brazilian reference sequences, and 5 HIV-1 Subtype C sequences as outgroup). The analyzed fragment corresponds to 891 bp (2262 to 3251 nt relative to HXB2 genome) and drug-resistance mutations positions were stripped.

**FIGURE 4:**
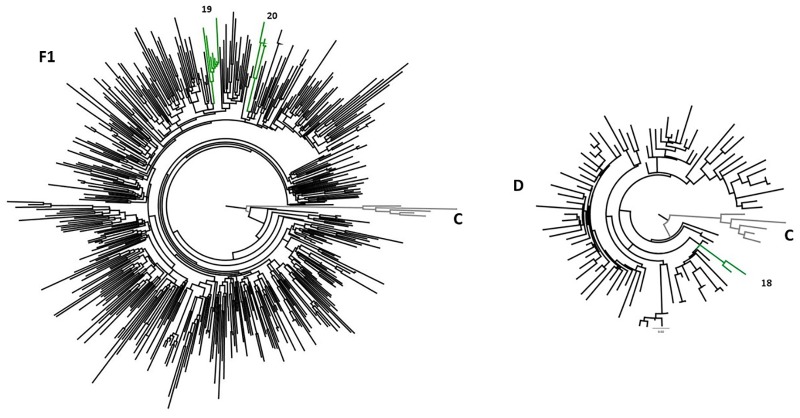
ML phylogenetic tree showing the transmission clusters among HIV-1 subtypes of D and F1 sequences. The confirmed HIV-1 transmission clusters were highlighted in green and numbered according to the previous grouping from Figure 1. All clusters present aLRT ≥ 0.90 and low mean pairwise genetic distances (≤4.5). The sub-subtype F1 analysis involved 493 PR/RT sequences (15 sequences from the present study, 467 Brazilian reference sequences, 6 non-Brazilian reference sequences, and 5 HIV-1 Subtype C sequences as outgroup). From subtype D analysis, 94 sequences were used as follows: 2 detected in the present study, 17 HIV-1 Brazilian reference sequences, 72 non-Brazilian reference sequences and HIV-1 Subtype C sequences as outgroup).

Twenty-four possible transmission clusters, including 57 individuals were identified according to the adopted criteria (aLRT > 90 in ML analysis). The clusters involved from two to five individuals and seventeen of them belong to HIV-1 subtype B, one to subtype D, three to sub-subtype F1 (Figure 2A) and three were recombinant forms being 2 BF1 and 1 BD (Figure 2B). The inclusion of a huge number of reference sequences enabled reinvestigation by ML of the transmission clusters, in combination with the criteria of presenting high aLRT support and low mean genetic distance, allowed us to depict twenty previously identified possible transmission clusters from pure HIV-1 subtypes B, D, and F1. The possible transmission clusters 1c, 3, 10, and 21 were not confirmed. Some of the originally detected clusters remained with the same configuration (2,8,11,13,16, and 18); meanwhile, some of them presented a new shape. In the Cluster numbers (1, 4, 5, 9, 15, 17, 19, and 20) some Brazilian reference sequences clustered together to ours. We also verified that some sequences were excluded from the original possible clusters (1, 3, 6, 9, 10, 12, and 14). The possible clusters 1 and 6 give rise to two new transmission clusters (1a,b and 6a,b). The original possible clusters (Figure 2A,B) and the confirmed transmission clusters (Figure 3, 4) were summarized in Table 3. Since the clusters BD (22) and BF1 (23 and 24) were unique recombinant forms, we did not perform an additional ML phylogenetic tree.

**Table 3:** Cluster confirmation of cART-naïve HIV-1 sequences according to aLRT and genetic distance.

Figure 2			Figure 3				Transmission cluster confirmation
	
Sequence	Cluster number	aLRT	Sequence	Cluster number	aLRT	Genetic distance	
**Subtype HIV-1 B**							
HSH645	1	0.98	HSH645	1a	0.99	1.8	Confirmed
HSH684			HSH684				
HSH876			BRMS57				
HSH878			HSH878	1b	0.93		Confirmed
HSH543			HSH876			3.5	
HET220			BRMS171				
HET673			HSH543	1c	0.9	6.0	Not confirmed
HET075			HET220				
			HET673	not confirmed			
			HET075	not confirmed			
HET141	2	0.95	HET141	2	0.94		Confirmed
HET642			HET642			4.2	
HET737			HET737				
HET362	3	0.91	HET362	3	0.93	5.8	Not confirmed
HET622			HET622				
HET219			HET219	not confirmed			
HSH187	4	0.93	HSH187	4	0.95	3.4	Confirmed
HSH595			HSH595				
HET607			BRMS58				
			BRMS14_10				
			HET607				
HSH152	5	1	HSH152	5	1		Confirmed
HSH430			HSH430			3.9	
			BRMS40				
HSH408	6	0.92	HSH408	6a	1	1.3	Confirmed
HSH862			HSH862				
HSH871			HSH871	6b	0.99	1.4	
HET850			BRMS97				Confirmed
			BRMS99				
			HET850	not confirmed			
HET671	7	1	HET671	7	1	2.6	Confirmed
HET438			HET438				
HET517	8	1	HET517	8	1	3.9	
HET797			HET797				Confirmed
HSH703			HSH703				
HET562	9	0.93	HET562	9	0.96	3.6	
HET188			BRMS55				Confirmed
			BRMS05				
			HET188	not confirmed			
HSH143	10	0.93	HSH143	not confirmed			Not confirmed
HET437			HET437	not confirmed			
HSH239	11	0.92	HSH239	11	0.95		Confirmed
HSH066			HSH066			4.4	
HET694			HET694				
HSH881	12	0.96	HSH881	12	0.93	3.3	Confirmed
HET249			HET249				
HET814			HET814	not confirmed			
HSH874			HSH874	not confirmed			
HET367			HET367	not confirmed			
HET757	13	1	HET757	13	1	1.3	
HSH867			HSH867				
HSH875			HSH875				Confirmed
HSH716			HSH716				
HSH717			HSH717				
HSH53	14	0.98	HSH53	14	0.99	1.9	Confirmed
HSH94			HSH94				
HSH240			HSH240	not confirmed			
HET111	15	0.98	HET111	15	0.96	3.6	
HSH109			HSH109				Confirmed
			MS34				
HET38	16	0.98	HET38	16	0.94	3.7	Confirmed
HET510			HET510				
HET462	17	1	HET462	17	0.98	3.4	Confirmed
HET631			HET631				
HET801			HET801				
			MS02				
			MS46				

**Figure 2**			**Figure 4**				**Transmission cluster confirmation**
	
**Sequence**	**Cluster number**	**aLRT**	**Sequence**	**Cluster number**	**aLRT**	**Genetic distance**		

**Subtype D**							
HET463	18	1	HET463	18	1	3.4	Confirmed
HSH761			HSH761				
**Sub-subtype F1**							
HSH006	19	1	HSH006	19	0.97	4.0	Confirmed
HSH264			HSH264				
HSH265			HSH265				
HSH387			HSH387				
HSH534			HSH534				
			BR07SP153				


HET256	20	0.99	HET256	20	1	3.6	Confirmed
HET318			HET318				
HET361			HET361				
			BRMS38				


HET140	21	0.92	HET140	21	1	4.8	Not confirmed
HSH739			HSH739				
HET796			HET796				
			BRGO4074				
			BRGO6051				
**Recombinant BD**							
HET45	22	1			
HET46					
**Recombinant BF**							
HET643	23	1			
HET122					
HET483					
HET793	24	0.99			
HET353					


*HET: sample obtained from heterosexual individual; HSH: sample obtained from men who have sex with men.*

All subtype B sequences were classified as pandemic B. Among subtype B confirmed clusters, twelve (12/17; 70.6%) had more than two sequences, and five (5/17; 29.4%) were composed of two sequences. Five clusters comprised MSM samples of this study with or without other Brazilian sequences (clusters 1a, 1b, 5, 6a, and 14), four with HET samples (clusters 2, 7, 16, and 17), six, mixed HET, and MSM sequences (clusters 4, 8, 11, 12, 13, and 15). Two clusters (6b and 9) were formed by one sequence from our study and two other Brazilian sequences from MS state, retrieved from Genbank (Table 3).

Individuals from ten clusters of subtype B were positive for lifetime syphilis and/or Hepatitis B and C infections. Four (4/17; 23.5%) contained sequences with TDRM, and two of them (clusters 1b and 4) were composed by MSM sharing the same TDRM. Cluster 1b included two MSM who had a history of Treponema pallidum infection and K103N mutation, and one of them reported being a sex worker and bisexual. Cluster 4 grouped two sequences from MSM (HSH187 and HSH595), one from a male HET, and sequences BRMS58 and BRMS14_10, both from males (da Silveira et al., 2012), all of them had the V75M substitution, associated with resistance to NRTI inhibitors.

Samples belonging to non-B subtypes were grouped into three clusters (Figure 4). Two of them (19 and 20), belonging to F1 subtype, contained more than two samples. The cluster 19 contained five sequences from MSM, three of which reported the use of illicit drugs and two were positive for syphilis (anti-*T. pallidum*). Additionally, cluster 19 also grouped a sequence from São Paulo (Brígido et al., 2011). The two samples characterized as subtype D clustered together (cluster 18).

## Discussion

This phylogenetic study combined detailed clinical and epidemiological data, providing valuable data for surveillance, which allowed the monitoring of HIV-1 variants, TDRM, and associations between sociodemographic characteristics and behavioral sexual groups. It is noteworthy that the study subjects were antiretroviral naïve, and therefore, they were not in virologic suppression at the time of sample collection. This fact, associated with unprotected sexual practices, a multiplicity of sexual partners and a history of sexually transmitted infections (STIs), may be crucial for the maintenance of high HIV transmission rates.

In this study, HIV-1 B subtype was identified in 67.3% of the isolates, followed by recombinant forms, subtypes F1, C, and D. This distribution reflects that found in most Brazilian regions (da Silveira et al., 2012; de Moraes Soares et al., 2014). The frequency of 13.3% (95% CI: 7.9 to 18.8%) of recombinant forms found in this study was similar to that found in previous studies conducted in Central Brazil (16.3% and 14.5%) (Stefani et al., 2007; da Silveira et al., 2012). The absence of the Caribbean non-pandemic subtype B (B_CAR_) differs from the previous study by Divino et al. (2016), where a frequency of 5.5% from B_CAR_ were detected in Mato Grosso do Sul. Previous studies conducted in a southern region of Brazil identified differences in the distribution of subtypes according to sex and exposure category (De Sa Filho et al., 2005; Dias et al., 2009). The present study is the first conducted in MS addressing this issue, and the lack of association herein can be justified by the high frequency of bisexual behavior (33.9%) reported by homosexual individuals from our cohort, suggesting that the differential transmission of subtypes according to the exposure category is not restricted to the MSM.

In the present study, an intermediate prevalence (12.0%) of TDRM was found, according to the WHO classification (Bennett et al., 2009), which is higher than that found in Northern Brazil (1.0%) (dos Anjos Silva et al., 2016) and is consistent with those found in previous Brazilian studies using similar sequencing technologies (6.8% to 17.2%) (Brindeiro et al., 2003; De Sa Filho et al., 2005; Cardoso et al., 2009; Sprinz et al., 2009; Alencar et al., 2013; Pessôa et al., 2015; Arruda et al., 2018). Recently, among crack cocaine users in Central Brazil, a high prevalence of TDRM was found (58.3%). It is worth noting that only 12 HIV-positive individuals were investigated (Da Silva França et al., 2018).

Recently, one study using massive parallel sequences of Brazilian blood donors found an overall prevalence of TDRM in PR and RT regions of the HIV-1 *pol* gene of 44.5% (Pessôa and Sanabani, 2017). Insufficient data to evaluate the time of HIV-1 infection and conventional sequencing usage may have caused an underestimation of TDRM prevalence (Palmer et al., 2005; Jain et al., 2011; Mohamed et al., 2014). Besides, it has been reported that significant inequalities in access to treatment persists in Brazil, resulting in different impacts on mortality in some groups, such as non-white individuals, or those with poor formal education (Lima et al., 2018).

It is remarkable that 4.0% of virus isolates obtained in this study had multiple mutations that may further influence the response to treatment. K103N, the most frequent resistance mutation observed, is commonly related to decreased susceptibility to efavirenz and nevirapine and the V75M mutation was associated with lamivudine and/or stavudine use (NNRTI). Some studies point out that genotyping tests before initiation of cART for all patients could be cost-effective in Brazil (Sanabani et al., 2011; Luz et al., 2015). However, these tests are still available only to specific populations, such as serodiscordant partners and HIV infected pregnant women.

Although HIV prevalence among MSM increased beyond expectations in Brazil, no difference between TDRM prevalence in homosexuals and heterosexuals was observed in this study. This result may reflect trends of feminization and the increase in heterosexual transmissions observed in Brazil (Brasil, 2017). In contrast, (Bermúdez-Aza et al., 2011) found higher TDRM prevalence in MSM (21.4%) recruited in Brazil by respondent-driven sampling, a particular sampling technique for hard-to-reach populations. As a result, transmission networks of resistance variants may have been selected among these MSM, thus reflecting this prevalence. Due to the higher prevalence of HIV infection in MSM (Kerr et al., 2018) and transgender women in Brazil (Grinsztejn et al., 2017), pre-exposure prophylaxis is recommended by the Brazilian Ministry of Health, who have made efforts to implement it and suggest it may be cost-effective (Luz et al., 2018).

Transmission clusters are frequently defined by low genetic distance (1.0%-4.5%) within cluster sequences and high support phylogenetic clusters (Lewis et al., 2008; Bezemer et al., 2010), herein employing both resources we were able to determine nineteen transmission clusters. However, more recently, transmission network approaches have also been used to this purpose, such as HIV clustering (Wertheim et al., 2014), Cluster picker and Cluster Matcher (Ragonnet-Cronin et al., 2013).

Seventeen transmission clusters were confirmed among subtype B isolates, some of them grouped patients with co-infections. Further evidence suggests that unprotected sexual intercourse and the presence of STIs that cause ulcerative lesions such as syphilis play important roles as cofactors in HIV transmission (Lynn and Lightman, 2004; Karp et al., 2009). This emphasizes the importance of prevention and treatment interventions.

Preventive actions regarding HIV-1 transmission are needed to disrupt the network and to reduce the spread of TDRM, since 29.4% of the clusters (5/17) contained samples with TDRM. Two of these groups were sharing the same substitution, showing the possibility of transmission of resistance between these individuals. Therefore, since 2013 the Brazilian Health Ministry recommendation, following the WHO recommendation, established that all HIV infected individuals should start the treatment to accomplish viral suppression, this being an effective way to reduce the HIV transmission (Brasil, 2013).

Clusters containing sequences from individuals with different sexual behaviors, including homosexual and bisexual contacts, were found in HIV-1 B (clusters 4, 8,11,12, 13, and 15) and D subtypes (cluster 18). Thus, factors such as being a sex worker, having multiple sexual partners, inconsistent condom use, and bisexual behavior may increase exposure to resistant HIV-1 isolates, both in heterosexual and homosexual networks.

The detection of clusters containing Brazilian samples from previous studies in Central-Western and Southeastern Brazil (Brígido et al., 2011; Cardoso et al., 2011; da Silveira et al., 2012) can be explained by the high mobility of the population, reinforcing the possibility of the spreading of infection despite great geographic distances, thus influencing local dynamics of diseases. Therefore, transmission networks and potential links with the different exposure categories should be further investigated in Brazil.

The study has some limitations. We interviewed all individuals face-to-face; consequently, risk behaviors may have been under-reported, leading to potential underestimation of associations with these variables and TDRM prevalence. Moreover, due to the study design, sample composition may not be representative of Campo Grande-MS epidemic and the absence of time of HIV-1 infection or diagnosis can portray an older epidemic. Even using a very limited number (1.4% from the total number of AIDS cases in Mato Grosso do Sul) of HIV-1 sequences from Mato Grosso do Sul, we were able to detect transmission clusters. However, we could not obtain detailed epidemiological information about the sequences from other Brazilian studies that were in some clusters. On the other hand, these findings enhance the understanding of the HIV-1 genetic characteristics, transmitted drug resistance, and transmission networks, as the research comprises not only individuals with epidemiological features in common but also the spread of strains between homosexuals and heterosexuals.

We highlight the urgent need for increased transmission monitoring of antiretroviral-resistant isolates, aiming for the selection of more effective therapeutic regimens, viral suppression, and hence the interruption of HIV-1 transmission networks. Improved understandings of risks, including potential linkages between sexual exposures among MSM, may contribute to designing preventive interventions and for improving HIV surveillance regarding TDRM in the largest country in Latin America.

## Author Contributions

MLG and AM-C conceived the presented idea. TT, TFL, MLG, and AM-C discussed the results and wrote the manuscript. TT, SF, GC, and GR collected blood samples and also performed DNA extraction. AL provided medical support. TT, TFL, and MLG performed the experiments. TT, TFL, MLG, and AM-C analyzed the data. All the authors contributed to the final version of the manuscript.

## Conflict of Interest Statement

The authors declare that the research was conducted in the absence of any commercial or financial relationships that could be construed as a potential conflict of interest.

## Footnotes

1 https://www.hiv.lanl.gov
